# Factors Influencing Drug Prescribing for Patients With Hospitalization History in Circulatory Disease–Patient Severity, Composite Adherence, and Physician-Patient Relationship: Retrospective Cohort Study

**DOI:** 10.2196/59234

**Published:** 2024-12-06

**Authors:** Tomoyuki Takura, Hiroyoshi Yokoi, Asao Honda

**Affiliations:** 1 Department of Health Care Services Management Nihon University School of Medicine Tokyo Japan; 2 Department of Healthcare Economics and Health Policy Graduate School of Medicine The University of Tokyo Tokyo Japan; 3 Cardiovascular Center, Fukuoka Sanno Hospital International University of Health and Welfare Fukuoka Japan; 4 Saitama Prefecture Institute of Public Health Saitama Japan

**Keywords:** medication adherence, drug prescription switch, generic drug, logistic model, long-term longitudinal study, patient severity, systolic blood pressure, serum creatinine, aging, big data

## Abstract

**Background:**

With countries promoting generic drug prescribing, their growth may plateau, warranting further investigation into the factors influencing this trend, including physician and patient perspectives. Additional strategies may be needed to maximize the switch to generic drugs while ensuring health care system sustainability, focusing on factors beyond mere low cost. Emphasizing affordability and clarifying other prescription considerations are essential.

**Objective:**

This study aimed to provide initial insights into how patient severity, composite adherence, and physician-patient relationships impact generic switching.

**Methods:**

This study used a long-term retrospective cohort design by analyzing data from a national health care database. The population included patients of all ages, primarily older adults, who required primary-to-tertiary preventive actions with a history of hospitalization for cardiovascular diseases (*ICD-10* [*International Statistical Classification of Diseases, Tenth Revision*]) from April 2014 to March 2018 (4 years). We focused on switching to generic drugs, with temporal variations in clinical parameters as independent variables. Lifestyle factors (smoking and drinking) were also considered. Adherence was measured as a composite score comprising 11 elements. The physician-patient relationship was established based on the interval between physician change and prescription. Logistic regression analysis and propensity score matching were used, along with complementary analysis of physician-patient relationships, proportion of days covered, and adherence for a subset of the population.

**Results:**

The study included 48,456 patients with an average follow-up of 36.1 (SD 8.8) months. The mean age was 68.3 (SD 9.9) years; BMI, 23.4 (SD 3.4) kg/m^2^; systolic blood pressure, 131.2 (SD 15) mm Hg; low-density lipoprotein cholesterol level, 116.6 (SD 29.3) mg/dL; hemoglobin A_1c_ (HbA_1c_), 5.9% (SD 0.8%); and serum creatinine level, 0.9 (SD 0.8) mg/dL. Logistic regression analysis revealed significant associations between generic switching and systolic blood pressure (odds ratio [OR] 0.996, 95% CI 0.993-0.999), serum creatinine levels (OR 0.837, 95% CI 0.729-0.962), glutamic oxaloacetic transaminase levels (OR 0.994, 95% CI 0.990-0.997), proportion of days covered score (OR 0.959, 95% CI 0.948-0.97), and adherence score (OR 0.910, 95% CI 0.875-0.947). In addition, generic drug rates increased with improvements in the HbA_1c_ level band and smoking level (*P*<.01 and *P*<.001). The group with a superior physician-patient relationship after propensity score matching had a significantly higher rate of generic drug prescribing (51.6%, SD 15.2%) than the inferior relationship group (47.7%, SD17.7%; *P*<.001).

**Conclusions:**

Although physicians’ understanding influences the choice of generic drugs, patient condition (severity) and adherence also impact this decision. For example, improved creatinine levels are associated with generic drug choice, while stronger physician-patient relationships correlate with higher rates of generic drug use. These findings may contribute to the appropriate prescription of pharmaceuticals if the policy diffusion of generic drugs begins to slow down. Thus, preventing serious illness while building trust may result in clinical benefits and positive socioeconomic outcomes.

## Introduction

### Background

Many high-income countries with mature health care systems are actively expanding the use of generic drugs [1,2]. However, as generic drug use reaches a certain level, growth may slow (eg, Japan: 71.2% in 2018, annual rate of 5.45%; 77.8% in 2020, annual rate of 1.15%) [3,4]. Therefore, additional strategies may be required to maximize the switch to generic drugs with the sustainability of the health care system in mind, focusing on factors other than the conventional low cost. Specifically, it is important not only to emphasize the affordability of generic drugs but also to clarify other prescription considerations. For further promotion, the biological quality of generic drugs must be ensured, and their long-term health economics must be evaluated. Furthermore, both medical practitioners and patients must have a good understanding of the clinical characteristics and health care economics of generic drugs. Addressing the concerns of all stakeholders is crucial for optimizing prescriptions. This discussion should particularly focus on patient factors, including intrinsic adherence and physician-patient relationships.

The factors influencing the prescription of generic drugs can be classified into 3 major categories: patients, physicians, and regulations. While many studies have focused on physicians and regulatory factors, majority of the research on physicians is based on questionnaire surveys assessing their trust and knowledge of generic drugs, particularly among doctors and pharmacists [5-13]. Regarding regulatory factors, numerous studies have discussed the socioeconomic significance of the low prices of generic drugs against the backdrop of the medical insurance system [7,10,11,14,15]. However, patient characteristics have received less attention; although some reports have analyzed these factors as part of treatment outcome evaluations, few studies have specifically focused on how patient characteristics influence the selection of generic drugs [16].

Another theme related to the study of the factors influencing the prescription of generic drugs is the scattered outcome evaluation. Regarding this topic, relatively more study designs tend to compare clinical outcomes and patient compliance (eg, the proportion of days covered [PDC]) between brand names and generic drugs [17-30]. In addition, the analysis may address therapeutic inertia, which is characteristic of this theme. Studies tend to target diseases, such as cardiovascular diseases (CVDs) [17-22] and pain syndromes, for which generic drugs have been introduced as an alternative for expensive brand-name drugs. In this light, research on the factors influencing generic drug prescriptions is closely related to medical innovation (drug discovery) and the sustainability of the health care system (health insurance).

### Aim of This Study

Many studies have investigated the biological quality and economic use of generic drugs. However, as mentioned earlier, research on patient characteristics as limiting factors in generic drug prescriptions is lacking. For instance, few studies have explored the impact of complex diseases (severity) and inherent adherence on the switch to generic drugs. Most existing research has used the PDC as a surrogate for adherence, and no studies have analyzed widespread adherence (overall health-related behaviors), including disease prevention. In addition, while the impact of physician attitudes on PDC has been reported [13], no reports related to the patient-physician relationship for PDC or generic drug rates have been found.

In this context, we previously published a study suggesting that the selection of generic drugs could improve long-term life prognosis and reduce medical costs, influenced by composite adherence using drug dispensing data based on doctors’ prescriptions [26]. In the previous study, multivariate analyses were conducted to identify factors affecting long-term changes in the prognosis and clinical indicators from both public benefit and economic efficiency perspectives. The findings indicated that patient severity plays a significant role in the decision to switch to generic drugs. In addition, it was hypothesized that the patient-physician relationship, grounded in adherence, also plays a role in generic drug switching. Therefore, this study aimed to verify these hypotheses.

Thus, we conducted a study focusing on patient characteristics, primarily older adults, as a factor influencing generic prescribing. The study aimed to provide preliminary insights into how patient severity, composite adherence, and the patient-physician relationship impact the switch to generic drugs.

## Methods

### Application of Findings From Data Science

This study is a continuation of our previous study [26] on the development of a validation and prediction model for the long-term prognostic impact of adherence on health care costs and clinical outcomes in circulatory diseases. This was a long-term longitudinal multicenter retrospective cohort study.

In a previous study, we developed a predictive model of integrated health care resource consumption (Adherence Score for Healthcare Resource Outcome [ASHRO]) that incorporated patient health behaviors and examined their association with clinical outcomes. Predictive models, including neural networks and random forest learning (artificial intelligence), were used in that study. Adherence, measured by the ASHRO score, was considered a broad concept encompassing moral and public interest perspectives [26]. This adherence score was also selected as a key factor in this study owing to the socioeconomic implications of drug prescriptions, which form the context of this research.

In the previous study, the examination of the basic model by machine learning was summarized as follows: Traditional empirical statistical methods, which involve obtaining a dataset with results, predictors, and fit coefficients, were not optimal for this exploratory study’s multivariate analysis on large samples. Consequently, we used machine learning techniques, specifically random forests, and K-fold cross-validation, to select and integrate explanatory variables and establish weights from the previous study.

Random forests are machine learning techniques used for classification and regression that can help minimize overfitting [27,28]. The advantage of medical big data is their ability to efficiently handle large samples with thousands of input variables. In addition, random forests can accommodate various data scales (eg, blood pressure and glomerular filtration rates have different normal ranges) and remain robust even when unrelated variables are included [29,30].

The study developed a basic prediction model for medical and long-term care costs using random forests. We included over 100 variables related to medical practices, clinical tests, and preventive activities as explanatory factors. Machine learning was used to evaluate parameter integration and feature importance by randomly selecting multiple sample sets and feature variables using the bootstrap method (sampling with replacement).

### Data Sources and Populations

#### Data Sources

A previous study that developed a composite adherence score used a national health care database (Kokuho Database) that individually links health checkups, health insurance claims, and long-term care insurance data. This study used the same data source, and extracted a cohort from it.

The Kokuho Database is a large-scale repository that includes self-employed and retired individuals covered by National Health Insurance. It provides long-term medical and care insurance data, with a personal collusion rate between medical and long-term care information exceeding 99%. The regions included in the database accounted for 6.1% of Japan’s total population. In addition to the demographic trends and social structures, the main conditions of medical and long-term care generally reflect the average level in Japan. The data were managed and analyzed as part of the Health Economics Big Data of the University of Tokyo.

Data from each patient, anonymized under a unified identification, included basic characteristics, medical expenses related to hospitalization, outpatient care, dispensing, and dentistry; diagnostic information; breakdown of medical treatments; frequency of medical examinations; hospitalization duration; nursing care costs and required care level; usage frequency and duration; health checkup guidance content; laboratory and biological test results; and the number of unhealthy behaviors. We excluded samples with missing data to address potential bias in the large dataset.

Although the long-term care insurance system operated by the Japanese government focuses primarily on long-term care, it also covers chronic care. Nursing care is a key indicator of “functional impairment” and is a crucial metric, particularly in geriatric research. The system classifies daily living abilities using the nursing care level, which measures the degree of care required [31]. This classification includes 7 categories: “two stages of support required” and “five stages of nursing required”; higher numbers indicate greater severity. These care categories were scored and analyzed in this study.

#### Data Populations

**Figure 1:**
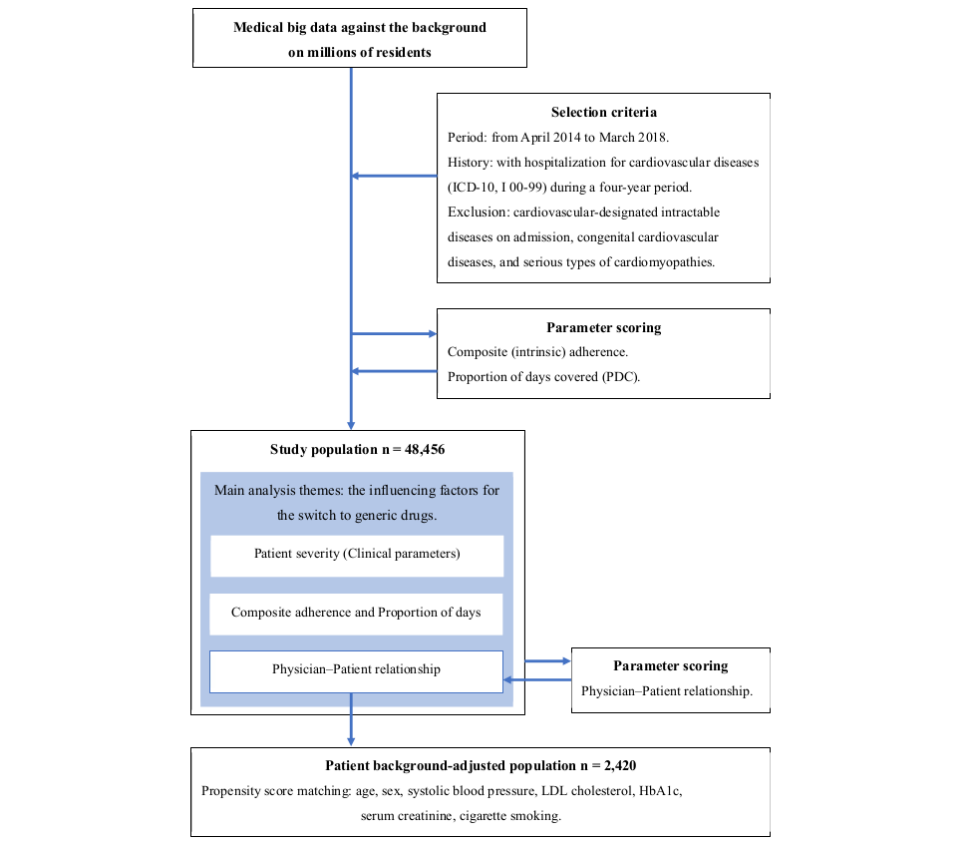
Flow chart of the study cohort setting. The propensity score method was used to compare the level of generic rates according to patient-physician relationship. LDL: low-density lipoprotein; HbA_1c_: hemoglobin A_1c_; ICD: International Classification of Diseases.

The study cohort comprised patients of all ages requiring preventive actions from first-to-tertiary levels, with a history of hospitalization for CVDs (*ICD-10* [*International Statistical Classification of Diseases, Tenth Revision*] codes I00-I99) from April 2014 to March 2018 (Figure 1) [32]. The cohort predominantly included older adults. This single-gate, multicenter retrospective observational study used real-world population data from an area with over 3 million residents. Exclusion criteria included cardiovascular intractable diseases upon admission, congenital CVDs, and serious types of cardiomyopathies [26,33].

As this was a longitudinal study, missing data at follow-up due to participants’ death or relocation were censored. Furthermore, we adjusted for patient background using the basic characteristics of sex and age, clinical indices of cardiac function, renal function, endocrine metabolism, and lifestyle habits, such as smoking, to reduce bias in the analysis of physician-patient relationships.

Sex differences in CVD are well-documented, with female generally having a lower incidence of ischemic heart disease than men [34]. In addition, coronary artery disease in female often develops later in life, with many cases involving cumulative risks, such as hypertension and dyslipidemia [35]. The population composition in this study reflected these trends.

### Definition of Key Indicators

#### Concept of Adherence Indicators

In our previous study, adherence was developed as a broad concept to discuss the behavioral changes in social groups and factors intrinsic to individual patients. Against this background, adherence is considered a composite measure. The 11 components comprising the final composite adherence (ASHRO) included indicators related to health promotion, prevention of disease severity, rational resource consumption behavior (moral hazard), medical attitude behavior, and public behavior (Multimedia Appendix 1) [26].

The following 11 indicators were calculated as the ratio of the difference between the mean value of the population and the mean value of each individual during the 1-year follow-up period after enrolment: number of health check-ups, units of rehabilitation intensity, number of guidance sessions, number of overlapping outpatient visits, clinical laboratory and physiological tests, inpatient days, number of outpatient visits, dispensing, PDC, and generic drug rate. This study used drug dispensing data based on doctors’ prescriptions.

Composite adherence (ASHRO) was scored while ensuring a significant correlation with risk factors, such as systolic blood pressure, serum creatinine, low-density lipoprotein cholesterol (LDL-C), and hemoglobin A_1c_ (HbA_1c_) levels, and the estimated glomerular filtration rate.

Overlapping outpatient services for the same diseases during the same period were similar. The generic drug rate is calculated as the proportion of prescriptions for generic drugs listed by the government, with the number of generic prescriptions as the numerator. The PDC was calculated based on the duration and continuity of prescriptions rather than individual medication adherence data. It is important to note that the PDC rate data reflect actual results, and the PDC score was incorporated into the ASHRO score. Each score serves a distinct purpose with different scales and interpretations.

#### Indicator of the Physician-Patient Relationship

In this study, we examined the relationship between physicians and patients. Although a questionnaire survey could be used to explain the relationship quantitatively, it was deemed unrealistic due to concerns about reliability from subjective judgments and the technical challenges or burden of integrating it with big data. Therefore, we extracted surrogate indices related to the relationship between doctors and patients from big data to prepare quantitative data matching actual clinical practice. Furthermore, we constructed a proxy index for trustworthy relationships using two elements: (1) change in the doctors or facilities for the same patient and (2) continuation of prescriptions at the same institution.

To examine this surrogate index, we proposed the following conditions hypotheses. First, a strong physician-patient relationship would be associated with fewer changes in physicians (eg, doctor shopping) [36]. Second, due to the characteristics of the cohort in this study, it was assumed that continuous (regular) medical care would be provided if the relationship was appropriate. Based on these hypotheses, we assessed the physician-patient relationship by evaluating the presence or absence of facility changes during the observation period and interval between consultations and classified the samples accordingly.

We categorized patients into 2 groups: those who experienced a change in facilities and those who did not, based on their visits for the same disease during the observation period. In addition, we considered information on the changes in the address. We labeled conditions with no change in the facility and a practice interval of 365 days or less as a superior relationship group (coded as 0) to indicate a strong physician-patient relationship. The other groups were considered as inferior relationship groups (coded as 1). The interval between treatments was set using a cutoff value derived from the receiver operating characteristic curve for a conservative assessment.

### Statistical Analysis

Logistic regression analysis was performed to examine the influence of the clinical indicators on the choice of generic drug prescriptions. We also performed a logistic regression analysis to examine the complementary factors in the physician-patient relationship. Mann-Whitney *U* test was used to compare the percentage of generic drug prescription choices by high and low physician-patient relationships, and multiple regression analysis was performed to determine the relationship between PDC rate and other factors.

**Figure 2:**
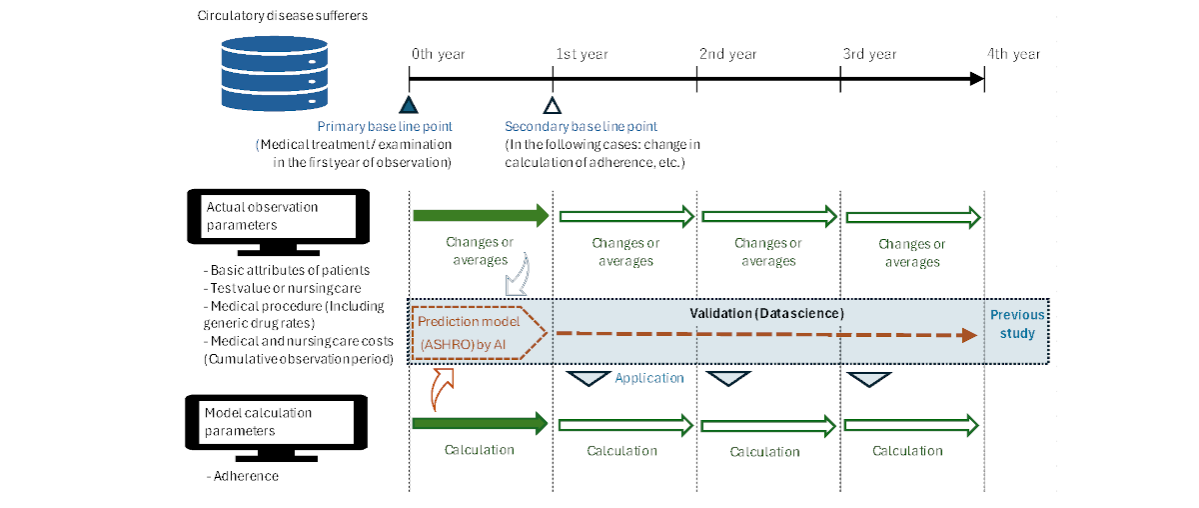
Timeline of this study and measurement methods for each parameter. This study was not an interventional study, so no fixed index time point was set. AI: Artificial intelligence; ASHRO: Adherence Score for Healthcare Resource Outcome.

The influence of the clinical indicators on the choice of generic drug prescriptions was organized based on the relationship between the displacement (annual mean value) of each indicator and the generic drug rate. Change was calculated as the change from the previous year’s value, either increasing or decreasing. The annual mean serum creatinine level was used as a surrogate because the progression mechanism was irreversible in the medium-term. The PDC and adherence indices were calculated based on 4 years of data; therefore, data on change were not used (Figure 2).

A comparison of the levels of generic rates by patient-physician relationship was performed for the generic switch factor, adjusted for patient background using propensity score matching (PSM). Propensity scores for each case were calculated, and a 1:1 matching method was applied to align the sample numbers and ensure the data distribution and balance between both groups. It is important to note that because this study aimed to assess the influence of actual measured severity (laboratory values) on prescription choice, we did not perform background adjustment using PSM was not performed in the overall basic analysis. Categorical variables are expressed as numeric values (%), and continuous variables are expressed as mean (SD). The statistical significance level was set at 5%. The probability of an outcome occurring was organized in terms of odds ratios (ORs). The software used was SPSS Statistics for Windows (version 27; IBM Corp).

In Japan, medical policy has encouraged the prescription of generic drugs, resulting in a steady annual growth (average annual rate: 4.27%) [4]. We also performed an analysis that considered this growth trend, adjusting for the displacement of generic drug use rate over time.

### Ethical Considerations

This study was approved by the Research Ethics Committee (2018167 N1: The Health Economics Big Data) of The University of Tokyo Hospital and involved strict data confidentiality in accordance with the Helsinki Declaration and Japanese Government’s Guidelines for Clinical Research Ethics and Reporting of Studies Conducted using the Observational Routinely Collected Data Statement.

## Results

The findings of this study are as follows. For reference, we compiled a summary (list) of all results in an additional file (Multimedia Appendix 2).

### Cohort Characteristics

A total of 48,456 patients were enrolled, with an average follow-up period of 36.1 (SD 8.8) months. The mean age was 68.3 (SD 9.9) years, and most patients were males (61.9%). At the baseline major health check-up, the BMI was 23.4 (SD 3.4) kg/m^2^, systolic blood pressure was 131.2 (SD 15) mm Hg, triglyceride level was 120.8 (SD 5.2) mg/dL, LDL-C was 116.6 (SD 29.3) mg/dL, HbA_1c_ was 5.9% (SD 0.8%), and serum creatinine was 0.9 (SD 0.8) mg/dL (Table 1).

**Table 1:** Cohort characteristics.

Parameter			Mean (SD)	Median (IQR)
**Sample**				
	Patients, n	48,456		—^a^
**Health check-up examination**				
	Age, years	68.3 (9.9)		69 (65-73)
	Male sex, n (%)	29,994 (61.9)		—^a^
**Physical examination**				
	Height, cm	160 (8.8)		160.3 (153.4-166.6)
	Weight, kg	60.0 (11.3)		59.5 (52.1-67.1)
	BMI, kg/m^2^	23.4 (3.4)		23.2 (21.1-25.3)
	Waist, cm	84.4 (9.3)		84.2 (78.5-90)
	Systolic blood pressure, mm Hg	131.2 (15)		130.5 (121.8-140)
	Diastolic blood pressure, mm Hg	75.7 (10.3)		75.3 (69.3-81.8)
**Lipid profile**				
	Triglycerides, mg/dL	120.8 (75.2)		103.3 (76-143.5)
	HDL^b^ cholesterol, mg/dL	59.4 (15.9)		57.5 (48-68.8)
	LDL^c^ cholesterol, mg/dL	116.6 (29.3)		116 (97-134.5)
**Kidney function**				
	Serum creatinine, mg/dL	0.9 (0.8)		0.8 (0.6-0.9)
	Serum uric acid, mg/dL	5.4 (1.4)		5.4 (4.5-6.3)
	eGFR^d^, mL/min/1.73 m^2^	69.2 (17.1)		69.6 (60.2-79.1)
**Blood sugar**				
	HbA_1c_^e^ (%)	5.9 (0.8)		5.7 (5.5-6.1)
Follow-up period, months			36.1 (8.8)	44 (25-48)

^a^—: not applicable.

^b^HDL: high-density lipoprotein.

^c^LDL: low-density lipoprotein.

^d^GFR: glomerular filtration rate.

^e^HbA_1c_: hemoglobin A_1c_.

The superior physician-patient relationship group comprised 10,332 patients. The mean age was 70.03 (SD 10.10) years for this group and 66.26 (SD 8.47) years for the inferior relationship group. The proportion of males was 67.15% in the superior relationship group and 60.44% in the inferior relationship group.

After PSM, no statistically significant differences were observed between the 2 patient-physician relationship groups (inferior and superior groups): mean change in the systolic blood pressure was 8.9 (SD 5.6) mm Hg in the inferior group and 8.5 (SD 5.5) mm Hg in the superior group (*P*=.14), and the mean serum creatinine levels were 0.6 (SD 0.8) mg/dL and 0.7 (SD 1.1) mg/dL in the inferior and superior groups, respectively (*P*=.13) during the observation period (Table 2). The sample size was 1210 in each group.

**Table 2:** Adjusted backgrounds of physician-patient relationship groups using propensity score matching.

Parameter	Inferior group	Superior group	*P* value
Sample, n	1210	1210	—^a^
Age (year), mean (SD)	70.2 (6.6)	70.2 (6.8)	.44
Sex^b^, male, n (%)	816 (67.4)	836 (69.1)	.41
Systolic blood pressure, mm Hg, mean (SD) change per observation period	8.9 (5.6)	8.5 (5.5)	.14
LDL^c^ cholesterol, mg/dL, mean (SD) change per observation period	7.8 (5.3)	7.4 (5.1)	.09
HbA_1c_^d^, % mean (SD) change per observation period	0.3 (0.2)	0.3 (0.2)	.28
Serum creatinine, mg/dL, mean (SD) per observation period	0.6 (0.8)	0.7 (1.1)	.13
Cigarette smoking^e^, binary, mean (SD) per observation period	0.2 (0.3)	0.2 (0.3)	.73

^a^—: not applicable.

^b^Sex: binary (male; 1, female; 2).

^c^LDL: low-density lipoprotein.

^d^HbA_1c_: hemoglobin A_1c_.

^e^Cigarette smoking: binary (presence; 0, existence; 1).

### Clinical Factors Influencing the Switch to Generic Drugs

**Figure 3:**
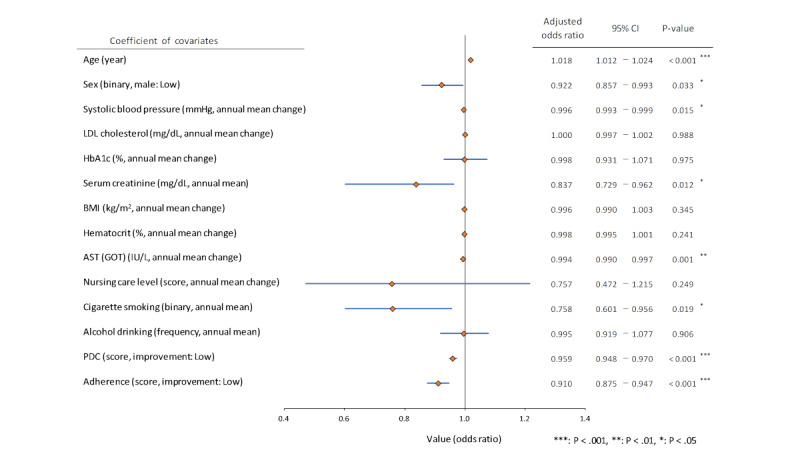
Clinical factors that influence the switch to generic drugs: logistic regression analysis. Nursing care is an indicator (degree of care) of the nursing care insurance system operated by the Japanese government. The level of nursing care is an index of the degree to which the older adults require nursing care. Depending on the physical and mental condition of the person being cared for, the condition will be classified into one of seven categories: “two stages of Support Required” or “five stages of Nursing Required”; the higher the number, the more severe the condition. Sex: binary (male: 1, female: 2), nursing care level: score (3 levels), cigarette smoking: binary (presence; 0, existence; 1), alcohol drinking: frequency (3 levels). LDL: low-density lipoprotein; HbA_1c_: hemoglobin A_1c_; BMI: body mass index; AST (GOT): aspartate aminotransferase (glutamic oxaloacetic transaminase); PDC: proportion of days covered.

Logistic regression analysis was performed by switching to a generic drug as the variable and various clinical parameters related to CVD as independent variables. Significant predictors included systolic blood pressure (OR 0.996, 95% CI 0.993-0.999; *P*<.05), serum creatinine (OR 0.837, 95% CI 0.729-0.962; *P*<.05), aspartate aminotransferase (OR 0.994, 95% CI 0.990-0.997; *P*<.01), PDC score (OR 0.959, 95% CI 0.948-0.970; *P*<.001), and adherence (OR 0.910; 95% CI 0.875-0.947; *P*<.001), all of which demonstrated significant associations with generic drug switching (Figure 3). Smoking also significantly influenced generic drug switching (OR 0.758; 95% CI 0.601-0.956; *P*<.05). No significant differences were observed in the LDL-C and HbA_1c_ levels between the groups.

The analysis corrected the rising trend in the generic drug use rate in Japan; thus, the impact of each parameter did not change compared with the previously mentioned OR results (systolic blood pressure, 0.997 and serum creatinine level, 0.878).

**Figure 4:**
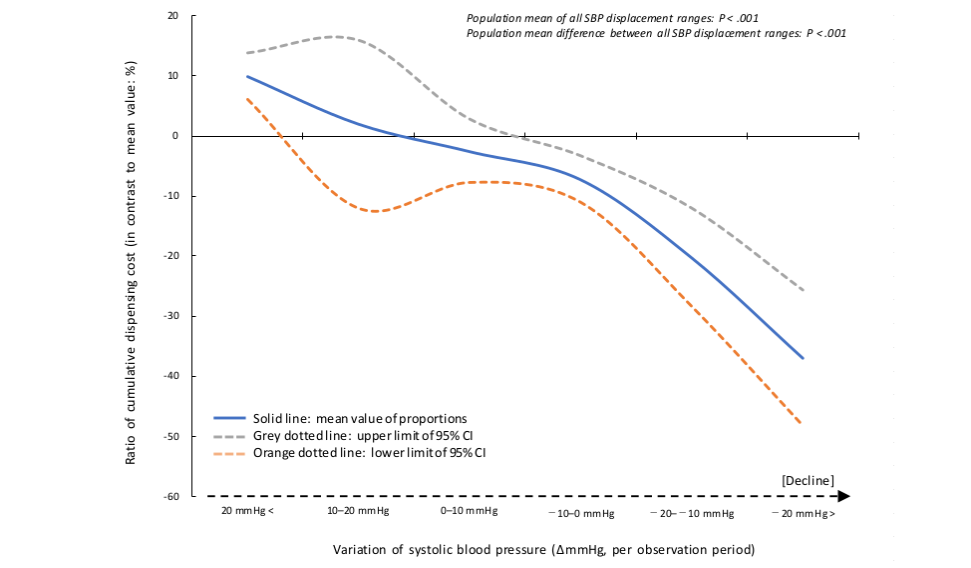
Relationship between systolic blood pressure variation and dispensing cost (percentage to mean value) SBP: systolic blood pressure.

**Figure 5:**
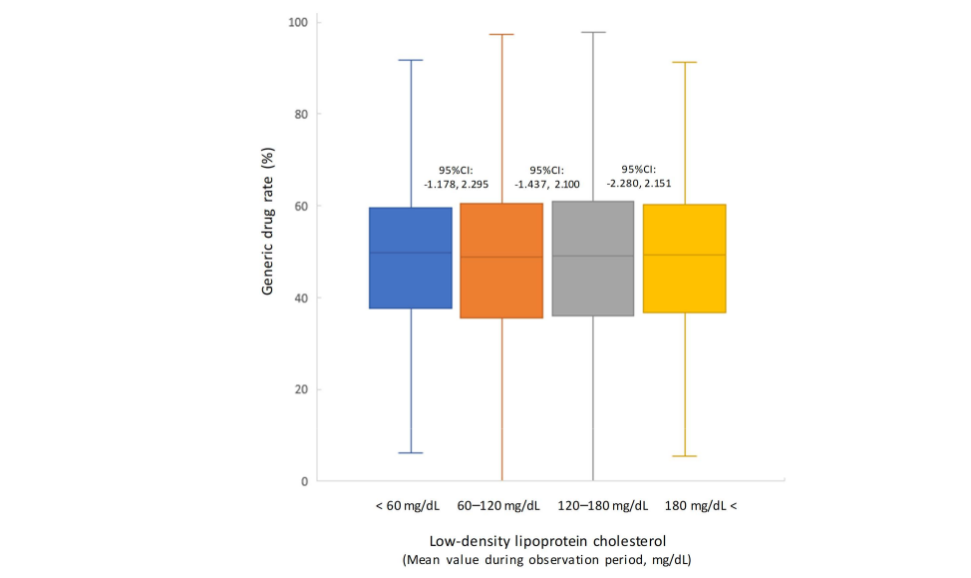
Generic drug rates at different mean low-density lipoprotein levels. Groups were compared using the Mann–Whitney U test, and no significant differences were detected. LDL-C: low-density lipoprotein cholesterol; NS: not significant.

**Figure 6:**
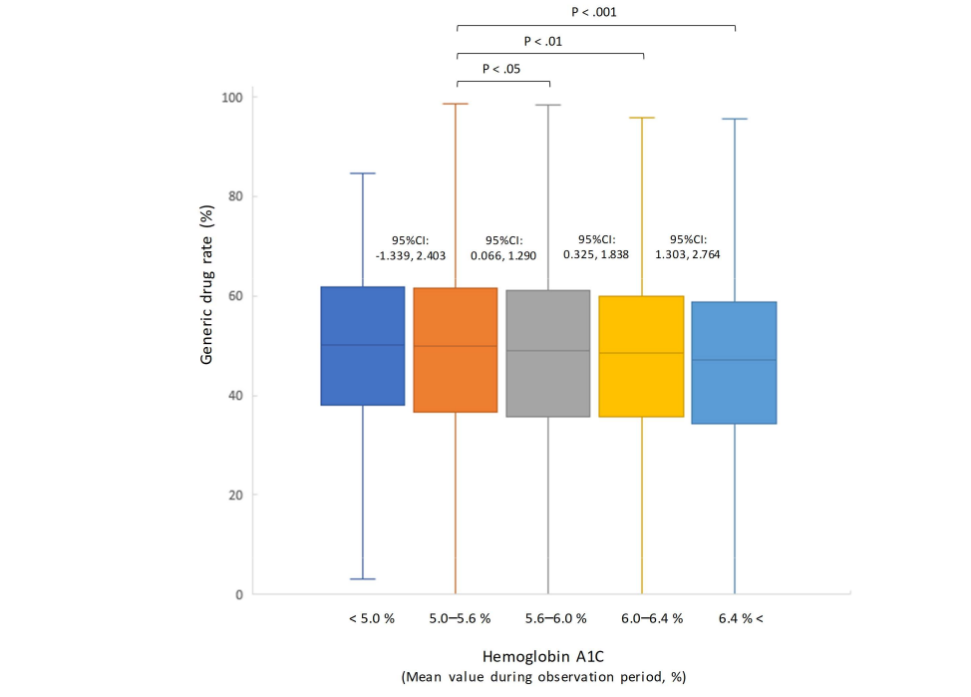
Generic drug rates at different mean hemoglobin A_1c_ levels. Groups were compared using the Mann–Whitney U test.

We organized the relationship between the amount of displacement and cost of dispensing (a surrogate for generic drug rates: integral of the unit price and quantity of the prescription drug) to complement the results for the OR of systolic blood pressure. The results showed a statistically significant reduction in dispensing costs with a decrease in blood pressure (population mean difference between all pressure displacement ranges: *P*<.001; Figure 4). For reference, we analyzed the generic drug rate according to LDL-C level bands. No significant differences were observed between the normal LDL-C level band and the other bands (Figure 5). In addition, we analyzed the generic drug rates using the HbA_1c_ level band. A significant decrease was observed between the normal LDL-C level band and other bands (HbA_1c_: 5%-5.6% vs 6%-6.4%, generic drug rate: 48.5% vs 47.4%; *P*<.01; Figure 6).

### Physician-Patient Relationship and PDC as Adherence Indicators

**Figure 7:**
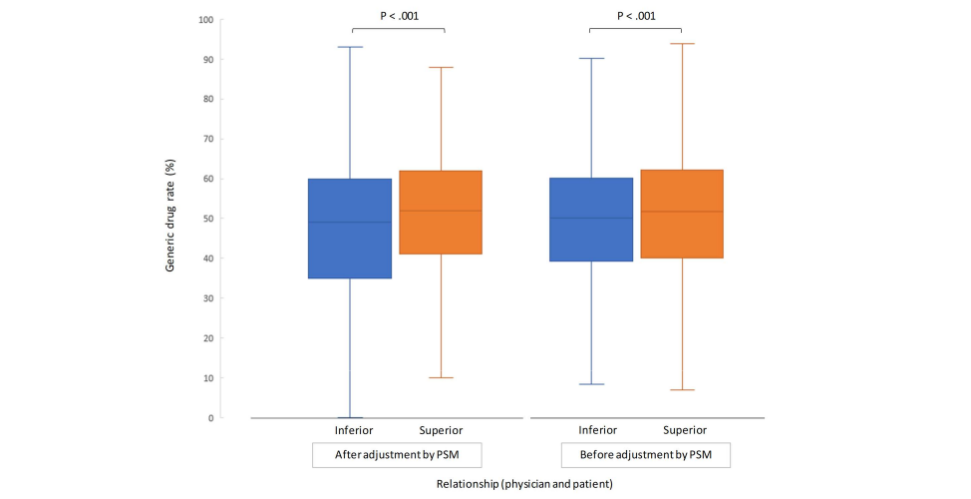
Generic drug rate according to the physician–patient relationship after adjustment for patient background. Groups were compared using the Mann–Whitney U test. PSM: propensity score matching.

When generic drug rates were sorted by physician-patient relationship after PSM, the superior relationship group had a significantly higher generic drug rate than the inferior relationship group (51.6%, SD 15.2% vs 47.7%, SD 17.7%; *P*<.001; Figure 7). The treatment interval cutoff value applied to the physician-patient relationship grouping (365 days) was a conservative estimate, as confirmed by the cutoff value derived from the receiver operating characteristic curve (494 days; area under the curve, 0.848; 95% CI 0.840-0.856; *P*<.001).

Multiple regression analysis with the PDC rate as the dependent variable revealed that an increase in the physician-patient relationship (standard partial regression coefficient:–0.254, *P*<.001) was a statistically significant factor for improving PDC (Table 3). The regression model (multiple regression equation, dependent variable: PDC rate) was statistically significant (*P<*.001, *df:* 10531).

**Table 3:** Analysis of factors affecting the proportion of days covered rate: multiple regression analysis.

Parameter	Standard partial regression coefficient	*F* test value (*df*=10531)	VIF^a^	95% CI (partial regression coefficient)	*P* value
Relationship (binarization)^b^	–0.254	733.169	1.04	–0.018 to –0.016	<.001
Age (year)	0.136	214.004	1.03	0.000-0.001	<.001
Sex (binarization)^c^	–0.020	4.787	1.00	–0.050 to 0.003	.029
Constant term	—^d^	19.152	—^d^	0.006-0.016	<.001

^a^VIF: variance inflation factor.

^b^Relationship: binary (superior: 0, inferior: 1).

^c^Sex: binary (male: 1, female: 2).

^d^—: not applicable.

Logistic regression analysis with the physician-patient relationship as the dependent variable suggested that adherence (ASHRO: OR 1.025; 95% CI 1.021-1.029; *P*<.001) was a statistically significant factor in improving the relationship (Table 4). Age tended to improve the PDC rate (standard partial regression coefficient: 0.136, *P*<.001, Table 3) and physician-patient relationship (OR 0.982; 95% CI 0.977-0.986; *P*<.001; Table 4). The regression model (logistic regression, dependent variable: relationship) was statistically significant (*P*<.001).

**Table 4:** Analysis of the effect of attribute factors on physician-patient relationships logistic regression analysis.

Parameter	Standard partial regression coefficient	Standard error	Odds ratio (95% CI; partial regression coefficient)	*P* value
Age (years)	–0.064	0.002	0.982 (0.977-0.986)	<.001
Sex (binarization)^a^	0.024	0.044	1.145 (1.050-1.249)	.002
Adherence (score, improvement: low)	0.104	0.002	1.025 (1.021-1.029)	<.001

^a^Sex: binary (male: 1, female: 2).

### Supplementary Analysis: Analysis Focusing on the Nursing Care Level Required, Which is a Phenomenon of Aging

In the logistic regression analysis mentioned above, no statistically significant relationship was found between the displacement in the required nursing care level and switch to generic drugs for the study population, the majority of which were older adults. This finding is noteworthy as the level of nursing care is often associated with aging, thus indicating the need to explore whether declining daily functioning owing to aging influences pharmacotherapy choices and their socioeconomic implications.

**Figure 8:**
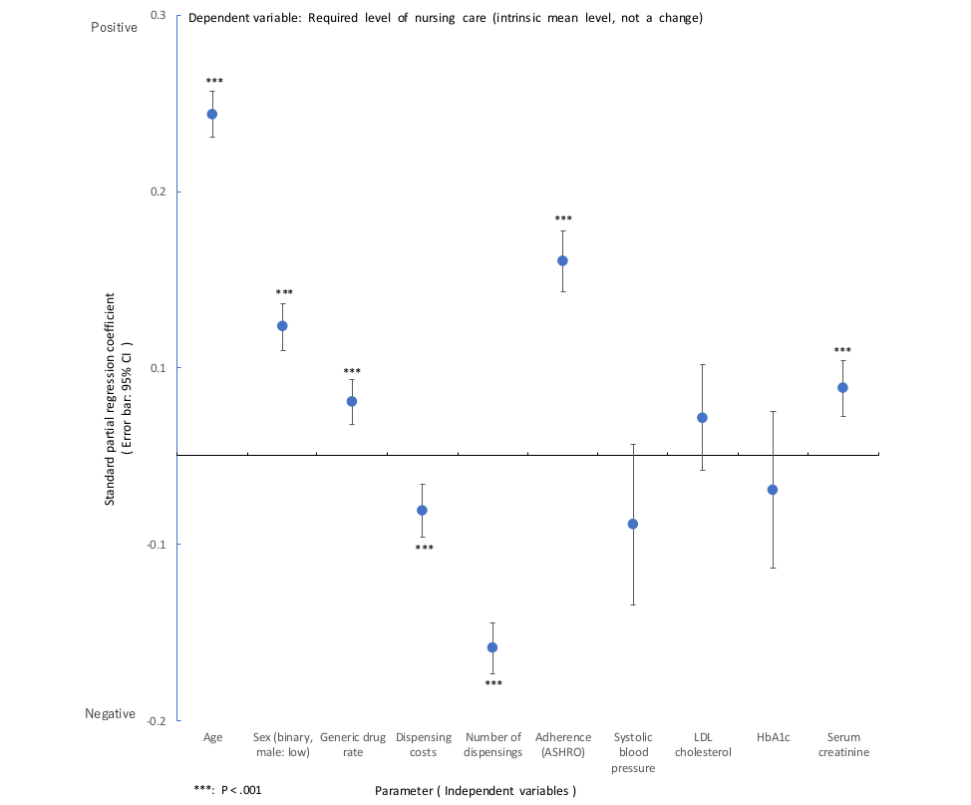
Analysis using the required level of nursing care (intrinsic mean level, not a change), which is a phenomenon of aging, as the dependent variable: multiple regression analysis. LDL: low-density lipoprotein; HbA_1c_, hemoglobin A_1c_.

In a multiple regression analysis using the intrinsic level of nursing care (as an aging phenomenon) as the dependent variable, the rate of generic drug use among older adults requiring nursing care was found to increase (standard partial regression coefficient, 0.051, 95% CI 0.043-0.059; Figure 8). While nursing care assessments focus on daily living functions and generally show little correlation with medical severity, cognitive function was included but did not reveal significant risk factors (eg, systolic blood pressure, –0.039, *P*=.09).

Furthermore, exploring the clinical and economic relationship between drug therapy and nursing care level, we observed that average nursing care levels tended to increase with a decrease in the dispensing frequency (standard partial regression coefficient, –0.109, 95% CI –0.123 to –0.094) and drug costs (standard partial regression coefficient, –0.031, 95% CI –0.045 to –0.016; Figure 8). Conversely, poor adherence (ASHRO) was associated with an increased nursing care level (standard partial regression coefficient, 0.110, 95% CI 0.093-0.127).

## Discussion

### Principal Findings

**Figure 9:**
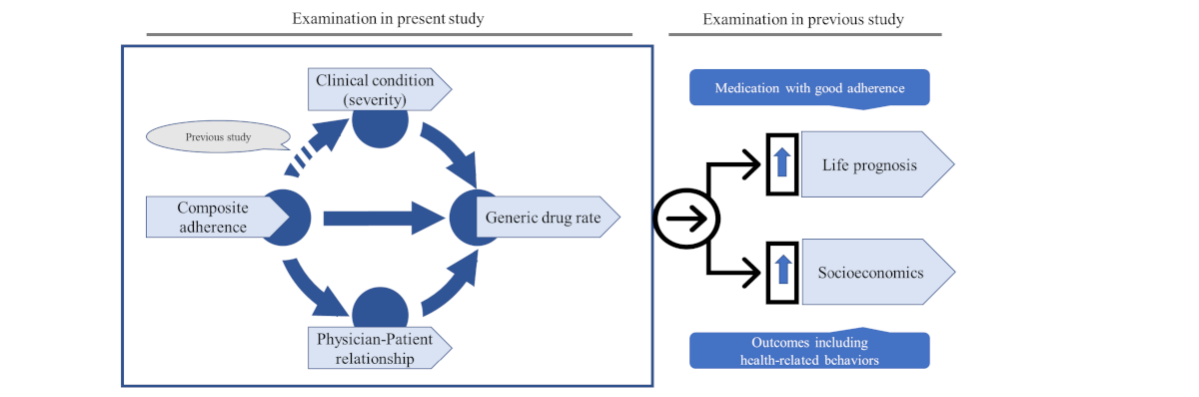
Summary of the findings and interrelationships of this study.

The results of this study demonstrated that improvements in the patient condition (severity) and intrinsic composite adherence were generally associated with increased generic drug switching. Furthermore, the rate of generic drug use tended to increase with improvement in the physician-patient relationship. The results also suggest that a better physician-patient relationship positively affects the PDC rate. Thus, enhancing comprehensive medical management and improving the medical environment is essential for further promotion of the appropriate selection of generic drugs. This includes fostering better relationships among stakeholders and increasing awareness among health care providers. Based on the above results and considerations, we organized the following mechanisms regarding the significance and social background of promoting generic drugs. Improvements in composite adherence improved the severity of patient illness and physician-patient relationships. Consequently, the number of prescriptions for generic drugs increased. Our previous study indicated that enhanced adherence positively impacts clinical outcomes and economic factors, such as life prognosis and medical costs [26]. Based on these conditions, it was speculated that generic drug selection would contribute to long-term clinical benefits and good socioeconomic impact (Figure 9). In addition, we speculate that the findings obtained in this study will contribute to the discussion on the prescription of brand-name drugs. However, future large-scale prospective studies are warranted to test this hypothesis.

As this study included patients with CVD who were hospitalized at least once, the cohort had a wide range of patient backgrounds. Therefore, the compositions of the prescribed medications were also widely distributed, not only for heart failure (CVD) but also for renal failure (chronic kidney disease [CKD]), endocrinology, and metabolism. For disease characteristics that repeat between the acute and chronic phases, we hypothesized that the sensitivity of health care providers to the severity (or complexity) of a patient’s condition would be high. The results of this study support this hypothesis. The presence of physician distrust and patient anxiety about generics was also considered terms of the diversity of diseases and drugs addressed in the study. In previous studies, there have been many reports investigating the confidence in or validation of clinical outcomes for generic drugs [16-25]. Scattered reports involving statins and warfarin showed no difference in the clinical outcomes and PDC between brand-name and generic drugs [18-21]. On the contrary, although only as a reference, the validation of generic drugs against pregabalin and gabapentin in the pain management of neuropathic pain showed that brand-name drugs were superior in improving PDC and reducing pain [23-25]. As described above, the evaluation of generic drugs varies according to the disease mechanism and drug class. This study did not delve deeply into individual drug characteristics, as it focused on organizing the overall trends from a long-term longitudinal analysis of a large real-world sample. This is one of the limitations of this study. Future studies elaborating on the patient characteristics and drug effect categories are warranted.

Many studies on the risk factors for CVDs (angina pectoris and myocardial infarction) have been conducted in Japan. Epidemiological studies in Japan and overseas have reported that hypertension is an independent risk factor not only for stroke but also for CVDs. For example, a 10-mm Hg increase in systolic blood pressure was associated with a 1.16- to 1.40-fold increase in the risk of developing or dying from ischemic heart disease [37,38]. In this study, the switch to generic drugs tended to increase considerably with an improvement in the systolic blood pressure, despite the OR to be small. Numerous epidemiological studies have shown that hypercholesterolemia is a risk factor for CVD [39]. Hyperlipidemia is the most important risk factor for CVDs. Japanese medical insurance targets the use of relatively new PCSK9 inhibitors in patients with statin intolerance. According to the analysis in this study, the OR of the LDL-C level for switching to a generic drug was neutral. Recent reports indicate that elevated LDL-C levels do not significantly affect the risk of death or vascular events [40,41]. A similar trend was observed in this study (Multimedia Appendix 3). Given the limited information available for deeper interpretation, future studies should extensively examine marker characteristics (such as diurnal variation), pharmacotherapy control status, and the evolution of target values in clinical guidelines. In addition, diabetes mellitus, a significant risk factor for CVD, should be considered in this context [42]. In contrast, the OR of the period change of HbA_1c_ levels for switching to a generic drug was neutral in this study. However, when the generic drug rate was analyzed according to HbA_1c_ level bands, a significant decrease was observed in the abnormal values than in the normal values. The estimated glomerular filtration rate and albumin-creatinine ratio were reported to be independent predictors of future acute kidney injury, CVD, CKD, nonfatal cardiovascular accidents, and death [43,44]. In this study, an improvement in the serum creatinine levels was associated with a marked trend toward switching to generic drugs. Aspartate aminotransferase displacement was also associated with the generic drug rate. Considering these findings comprehensively, improvements in the clinical indicators may influence the decision to switch to generic drugs.

Although the OR results showed statistical significance, there were also some indicators with extremely small numerical values. Caution should be exercised when interpreting these indicators clinically. The OR represents the degree of change (risk) of the target variable relative to the change of one unit of the independent variable. Considering the blood pressure index as an example, a change of 1 mm Hg in clinical practice is considered to have minimal clinical significance for a single patient because it may be affected by measurement errors and diurnal fluctuations. Typically, a change of 10 mm Hg is considered the standard for clinical discussion. Previous research has shown that an increase of 10 mm Hg increases the risk by 1.16 to 1.40 times [37,38]. In this analysis, we did not adjust the levels of the independent variables (eg, by a factor of 10), which may explain why some indicators showed smaller results. Therefore, when interpreted in terms of actual clinical practice, results with small ORs (indicating statistical significance) can be inferred to be significant in clinical practice. Particularly when discussing the representativeness of long-term fluctuations in large groups, even small ORs can be considered meaningful in the real world. Further examination of the data processing method for each analysis and the clinical usefulness of the results is desired through the development of new research.

In this study, it was inferred that many drugs had a high percentage of effects on the treatment outcomes due to medication compliance among the drugs studied. Therefore, patient adherence is an important factor in defining treatment outcomes. Given the characteristics of the study cohort, it was desirable to consider PDC as well as other health-related behavioral factors with regard to adherence. Particularly, the contribution of disease prevention interventions to improving long-term outcomes tended to be higher in populations with CVD and CKD associated with lifestyle-related diseases. Considering these factors, this study used the composite (intrinsic) adherence (ASHRO score). The results showed that improvements in the adherence scores, along with improvements in the levels of key clinical indicators, had a remarkable effect on the rate of generic drug use. Previous reports have suggested that improved PDC contributes to improved clinical outcomes [45-47]. The results of the analysis in this study suggest that composite adherence contributes to clinical outcomes since improved adherence scores improve PDC rates. In a previous study, the group with better ASHRO scores had better long-term life expectancy [26]. Incidentally, as adherence is an integrated index that also includes a PDC component, caution should be exercised when interpreting multivariate analyses involving PDC due to multicollinearity. Considering these points, this study has certain limitations in discussing causal inferences. Therefore, further verification of the representativeness of the data sources and the accuracy of the study design is desired.

A trial analysis of the relationship between physicians and patients was conducted to consider factors other than clinical indicators and adherence when examining factors leading to generic drug switching. Due to the difficulty in developing quantitative indicators of relationships, no previous studies have been conducted. A few studies have examined the effects of the level of information provision and medical personnel’s attitudes on PDC, and no clear effects have been found [11,13,46]. According to the results of this study, improving physician-patient relationships promoted generic drug rates. Composite adherence is positively related to this relationship. Therefore, promoting a good physician-patient relationship is expected to have a clinical effect. The PDC and this relationship tended to increase as patients’ age increased, which may be due to an increase in morbidities and a learning effect on patients [10]. Because this study was a long-term longitudinal observation, the physician-patient relationship indicator, which consisted of the visit interval component, included a time effect. Therefore, we cannot rule out the possibility that this effect may have influenced the aging trends described above. In this study, the physician-patient relationship was derived vicariously from the prescription entity, with medical ethics (including medical treatment institutional contracts) as a background. Therefore, this proxy indicator has limitations in discussing the psychological “relationship of trust” as a human relationship. In addition, the influence of confounding factors on analysis is also expected. Therefore, we believe that these findings should be interpreted with caution, and further studies are warranted to verify these mechanisms.

This study also considered the influence of government health care policies that promote the use of generic drugs. However, even after adjusting for current policies, the effect of clinical parameters on the rate of generic drug use remained largely unchanged. These findings may further encourage appropriate pharmaceutical prescribing, especially if the policy diffusion of generic drugs starts to slow. Prescribing generic drugs when appropriate could yield significant long-term socioeconomic benefits in clinical settings. For example, the argument above not only highlights clinical and economic benefits but also expands health policy options, potentially reducing social risks [48]. The findings of this study are particularly relevant for older adults requiring nursing care or end-of-life care, where multifaceted decision-making (shared decision-making) by various medical professionals is essential. The results of this study provide valuable insights into the realization of this objective.

The analysis revealed that patients requiring higher levels of nursing care tended to have higher prescription rates of generic drugs. This trend may seem contradictory to the earlier discussion on the relationship between medical severity and generic drug rates. However, examining the issue from different perspectives provides clarity. Two primary hypotheses could explain this phenomenon. First, long-term care insurance often involves higher out-of-pocket costs and services not covered by insurance. This may financially constrain individuals, especially after retirement, leading them to prefer lower-cost generic drugs [49]. Second, in nursing care facilities, which typically lack pharmacists, the preference for generic drugs may be driven by their lower prices and easier handling, reflecting the practical and financial constraints of managing long-term care services.

The interpretation that higher levels of nursing care are associated with increased generic drug prescriptions was supported by the general lack of a relationship between the unchanging average nursing care level and clinical indicators. This suggests that the level of nursing care and its degree of displacement do not necessarily align. One hypothesis is that the transition from medical institutions, where new drug prescriptions are more common, to nursing care facilities, where generic drugs are preferred owing to lower costs and ease of handling, could explain this discrepancy. The finding that drug costs decrease as the required care level increases and that adherence (measured by ASHRO) is related to the nursing care level supports this hypothesis. Specifically, drugs like donepezil hydrochloride, which is used to manage cognitive function, show a relationship between adherence and the nursing care level [50]. Despite this, the population with worsening nursing care had poorer adherence, highlighting that the dimensions of care level and medical severity may differ. Adherence appears to be more sensitive to medical severity, reflecting the complex interplay between care needs and medical management.

To our knowledge, this study is the first to focus on the patient-side factors involved in switching to generic drugs, using a long-term longitudinal cohort study design. This approach offers a novel perspective on how patient characteristics, adherence, and physician-patient relationships impact generic drug use over time. However, as shown in this study, the causal relationships related to the theme were complex. In particular, there was significant variability in changes in physical function and facility type among older adults. Therefore, we believe that leveraging data science is crucial for developing research strategies tailored to drug therapy for populations with such pathological characteristics. Moving forward, it would be beneficial to expand the application of reinforcement learning, particularly using prioritized experience replay. It is known for its effectiveness in addressing diverse problems, such as game theory and resource optimization, which holds promise for advancements in preventive medicine and social medicine.

This study has the following limitations. First, it did not delve deeply into the characteristics of individual drugs and the specific backgrounds of patients. Second, careful adjustment was not made for bias among the independent variables. Third, the physician-patient relationship was analyzed using proxy indicators. Consequently, the study’s design has limitations in terms of making causal inferences about this relationship.

### Conclusions

The patient condition (severity) and composite adherence (intrinsic) influenced the choice of generic drugs. Adherence tended to improve, and generic rates increased as physician-patient relationships improved. To further promote the appropriate selection of generic drugs, relationships with stakeholders must be improved, and patient awareness must be raised. Further development of methods to accurately evaluate adherence and relationships would be beneficial. The findings of this study suggest a novel approach for effectively promoting the use of both generic and brand-name drugs. However, the study possesses limitations affecting its ability to draw causal inferences.

## Appendix

Multimedia Appendix 1 Predictors of integrated medical and long-term care resource consumption.

Multimedia Appendix 2 List of the results of each analysis.

Multimedia Appendix 3 Relationship between 4-year average LDL level and 4-year cumulative all-cause mortality rate. Abbreviations: LDL, Low-density Lipoprotein Cholesterol Test: Spearman’s rank correlation coefficient.

## Abbreviations

ASHRO: Adherence Score for Healthcare Resource Outcome
CKD: chronic kidney disease
CVD: cardiovascular disease
HbA_1c_: hemoglobin A_1c_
ICD-10: International Statistical Classification of Diseases, Tenth Revision
LDL-C: low-density lipoprotein cholesterol
OR: odds ratio
PDC: proportion of days covered
PSM: propensity score matching
