# Supplementary material for: Factors Influencing Drug Prescribing for Patients With Hospitalization History in Circulatory Disease–Patient Severity, Composite Adherence, and Physician-Patient Relationship: Retrospective Cohort Study
Source: JMIR Aging. 2024 Dec 6;7:e59234. doi: 10.2196/59234 (PMC11662190; doi:10.2196/59234)
Supplement: Multimedia Appendix 1 [file aging_v7i1e59234_app1.pdf]

Multimedia Appendix 1. Predictors of integrated medical and long-term care resource consumption

**Health behavior**		**Composite adherence**	**No.**
Secondary prevention	→	Secondary prevention (Integrated)	1
Health check-ups, number per year		[a]	
Items of check-ups, number per year		[a]	
Tertiary prevention			
Rehabilitation intensity, units/year	→	Rehabilitation intensity	2
Guidance (e.g. lifestyle-related disease), number per year	→	Guidance	3
PDC, %	→	PDC	4
Overlapping outpatient service			
Outpatients visits, number per year	→	Overlapping outpatient visits	5
Clinical laboratory and physiological tests, number per year	→	Overlapping clinical laboratory and physiological tests	6
Medical attendance behavior	→	Medical attendance (Integrated)	7
Inpatient days, days per year		[b]	
Outpatients visits, number per year		[b]	
Dispensing, number per year		[b]	
Public behavior			
Generic drug rate, %	→	Generic drug rate	8
		(Complementary Indicators)	
		Age	9
		Sex	10
		Follow-up period	11

Components are integrated into the 'Composite adherence' index or the ' Secondary prevention' index through machine learning as indicated by 1 [a] and 7 [b] respectively.

Abbreviations: PDC, proportion of days covered; No, Number.

(Source: 26)
