# Supplementary material for: Factors Influencing Drug Prescribing for Patients With Hospitalization History in Circulatory Disease–Patient Severity, Composite Adherence, and Physician-Patient Relationship: Retrospective Cohort Study
Source: JMIR Aging. 2024 Dec 6;7:e59234. doi: 10.2196/59234 (PMC11662190; doi:10.2196/59234)
Supplement: Multimedia Appendix 2 [file aging_v7i1e59234_app2.pdf]

Multimedia Appendix 2. List of the results of each analysis

Part No.	Issue classification	Analysis items	Main findings	Figure / Table
2nd	Clinical influencing factors for the switch to generic drugs			
	a.	Clinical factors that influence the switch to generic drugs: logistic regression analysis	Logistic regression analysis found significant associations between generic switching and systolic blood pressure (odds ratio [OR]: 0.996, 95% confidence interval [CI]: 0.992–0.999), serum creatinine levels (OR: 0.837, 95% CI: 0.729–0.962), glutamic oxaloacetic transaminase levels (OR: 0.994, 95% CI: 0.990–0.997), PDC score, and adherence score.	Figure 3
	b.	Relationship between systolic blood pressure variation and dispensing cost	The results showed a statistically significant reduction in dispensing costs with the blood pressure decrease (population mean difference between all pressure displacement ranges: $P < .001$).	Figure 4
	c.	Generic drug rates at different mean hemoglobin A1C levels	Generic drug rates increased along with improvements in the HbA1c level band.	Figure 6
3rd	Physician–patient relationship and PDC as adherence indicators			
	a.	Generic drug rate according to the physician–patient relationship after adjustment for patient background	The group with a superior physician–patient relationship had a significantly higher rate of generic drug prescribing (51.6±15.2%) than the inferior relationship group (47.7±17.7%).	Figure 7
	b.	Analysis of factors affecting the PDC rate: multiple regression analysis	The physician-patient relationship was a statistically significant factor for improving PDC (standard partial regression coefficient: -0.254, $P<.001$).	Table 3
	c.	Analysis of the effect of attribute factors on physician-patient relationships: logistic regression analysis	Logistic regression analysis with the physician–patient relationship as the objective variable suggested that adherence [ASHRO] (OR: 1.025, 95% CI: 1.021–1.029, $P < .001$) was a statistically significant factor for improving the relationship.	Table 4
4th	Supplementary analysis: Analysis focusing on the nursing care level required, which is a phenomenon of aging			

a.	Analysis using the degree of care required, which is a phenomenon of aging, as the objective variable (multiple regression analysis)	As a result of performing a multiple regression analysis using the required level of nursing care, which is an aging phenomenon, as the objective variable, the rate of generic drugs in the population of older adults, requiring nursing care, was increasing (standard partial regression coefficient, 0.051, 95% CI: 0.043-0.059)	Figure 8
