# Supplementary material for: Factors Influencing Drug Prescribing for Patients With Hospitalization History in Circulatory Disease–Patient Severity, Composite Adherence, and Physician-Patient Relationship: Retrospective Cohort Study
Source: JMIR Aging. 2024 Dec 6;7:e59234. doi: 10.2196/59234 (PMC11662190; doi:10.2196/59234)
Supplement: Multimedia Appendix 3 [file aging_v7i1e59234_app3.pdf]

Multimedia Appendix 3. Relationship between 4-year average LDL level and 4-year cumulative all-cause mortality rate

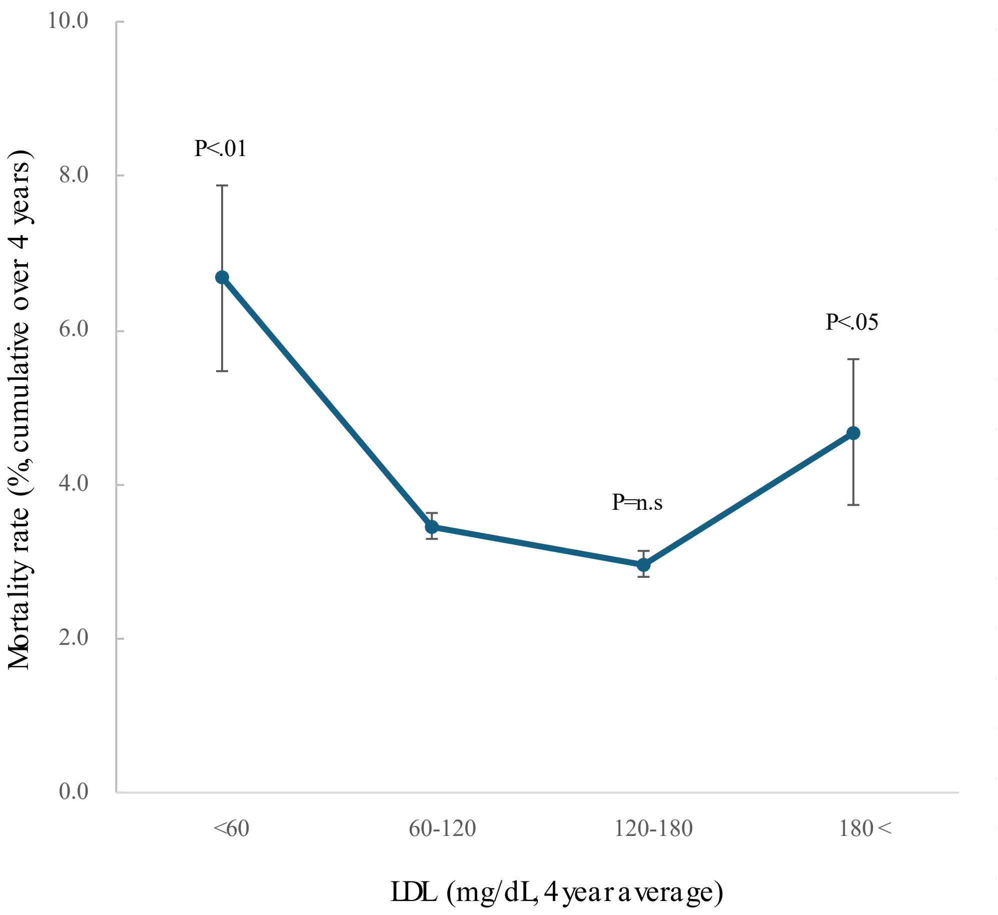

Abbreviations: LDL, Low-density Lipoprotein Cholesterol

Test: Spearman's rank correlation coefficient
